# Der Querschnittsbereich 14 „Schmerzmedizin“ an der Universität Leipzig – was wurde erreicht? Eine Analyse der Selbsteinschätzung der Studierenden vor, während und fünf Jahre nach Etablierung des Querschnittsbereichs

**DOI:** 10.1007/s00482-022-00665-7

**Published:** 2022-08-26

**Authors:** Gunther Hempel, Andreas Weissenbacher, Diana Becker-Rux, Swantje Mescha, Sebastian N. Stehr, Robert Werdehausen

**Affiliations:** 1 Klinik und Poliklinik für Anästhesiologie und Intensivtherapie, Universitätsklinikum Leipzig AöR, Liebigstr. 20, 04103 Leipzig, Deutschland; 2 Klinik für Anästhesiologie und Intensivmedizin, Universitätsklinikum Jena, Jena, Deutschland

**Keywords:** Befragung, Kompetenzbasierte Ausbildung, Medizinstudium, Curriculumsentwicklung, Prüfung, Undergraduate medical education, Survey, Competence-based education, Assessment, Curriculum development

## Abstract

**Hintergrund:**

Im Medizinstudium in Deutschland ist seit 2016 ein gesonderter Leistungsnachweis auf dem Gebiet der Schmerzmedizin zu erwerben. Bislang fehlen Untersuchungen über die Effekte dieser Einführung auf Kompetenzen von Studierenden im Themengebiet Schmerzmedizin.

**Ziel der Arbeit:**

Ziel war es herauszufinden, inwieweit die Einführung des Fachgebiets Schmerzmedizin als Querschnittsbereich 14 den Kompetenzerwerb der Studierenden im Bereich Schmerzmedizin gemessen über eine Selbsteinschätzung positiv beeinflusst.

**Material und Methoden:**

Die Entwicklung des longitudinalen Curriculums wurde mithilfe des Kern-Zyklus durchgeführt. Parallel erfolgte die Erstellung eines Fragebogens zur Selbsteinschätzung der Studierenden auf einer 5‑stufigen Likert-Skala bzgl. des eigenen Wissensstands und der Bedeutung schmerzmedizinischer Lehrinhalte. Die Befragungen wurden vor Einführung der Veranstaltungsreihe (2014), nach Abschluss der ersten Kohorte (2016) und 5 Jahre nach Einführung (2019) durchgeführt und mittels Kruskal-Wallis-Tests verglichen.

**Ergebnisse:**

Die Einführung des Curriculums hat zu einer signifikanten Verbesserung in relevanten Punkten geführt. So fühlen sich die Studierenden insgesamt besser auf die Behandlung von Schmerzpatienten vorbereitet (2,67 im Jahr 2014 vs. 3,18 im Jahr 2019). Teilaspekte wie die Erhebung einer Schmerzanamnese (3,63 vs. 4,10) oder die Erstellung eines Analgesieschemas (3,56 vs. 4,14) werden nun subjektiv deutlich besser beherrscht.

**Diskussion:**

Trotz erfreulicher Ergebnisse besteht in Teilbereichen noch Verbesserungspotenzial. Beispielsweise ist die Bewertung der Studierenden zur Frage nach der Vorbereitung auf die Behandlung von Schmerzpatienten noch nicht zufriedenstellend. Hier gilt es, das Curriculum mit Fokus auf die Kompetenzorientierung weiterzuentwickeln. Dabei können digitale Lehrformate ebenso integriert werden wie interprofessionelle Einheiten und Simulationspatienten. Zudem sind jedoch gleichzeitig auch die Prüfungsformate hin zu standardisierten praktischen Prüfungen weiterzuentwickeln.

**Zusatzmaterial online:**

Die Online-Version dieses Beitrags (10.1007/s00482-022-00665-7) enthält den Fragebogen zur Datenerfassung.

## Abstract (en)

**Background:**

The field of pain medicine was established as an obligatory subject area of medical schools in Germany in 2016. No prior study has evaluated the effects of this curricular change on students’ competences in the field of pain medicine.

**Objective:**

The aim of this study was to find out to what extent the introduction of the additional subject “pain medicine” positively influenced the students’ acquisition of competences measured via a self-assessment.

**Material and methods:**

A longitudinal and interdisciplinary curriculum for pain medicine was developed according to the current recommendations for curriculum development for medical education. In parallel, a questionnaire was created for the students’ self-assessment of their own level of knowledge and the importance of pain medicine teaching content on a 5-stage Likert scale. The surveys were conducted before the implementation of the curriculum (2014), directly after the first cohort finished (2016) and 5 years after the implementation (2019) and compared by Kruskal-Wallis test.

**Results:**

The implementation of the curriculum has led to significant improvement in relevant aspects. For example, students now feel better prepared overall for the treatment of pain patients (2.67 in 2014 vs. 3.18 in 2019). Individual sub-aspects such as taking a pain history (3.63 vs. 4.10) or drawing up an analgesia scheme (3.56 vs. 4.14) are now also subjectively better mastered.

**Conclusion:**

Even though the results are encouraging, there is further potential for improvement in some sub-areas. For example, the students’ rating regarding the question about their preparation for treating patients in pain is not yet satisfactory. Therefore, the curriculum should be developed further with a focus on competence orientation. Digital teaching formats can be integrated as well as interprofessional units and simulated patients. Additionally, the examination formats should be further developed towards standardized practical examinations.

Schmerzen zählen zu den häufigsten Gründen, aufgrund derer Patienten ärztliche Hilfe aufsuchen. Trotz dieser großen gesellschaftlichen Bedeutung war die Schmerzmedizin im Medizinstudium lange unterrepräsentiert und erst seit 2016 musste erstmalig ein gesonderter Leistungsnachweis für die Zulassung zum Staatsexamen erworben werden. An der Universität Leipzig wurde der Querschnittsbereich (QSB) 14 im Jahr 2014 etabliert. Mit der vorliegenden Arbeit sollte nun untersucht werden, ob die Anpassung des Curriculums seither zu einem spürbaren Wissenszuwachs der Studierenden im Bereich der Schmerzmedizin geführt hat.

## Hintergrund

Mit dem Ziel der Etablierung eines einheitlichen QSB für die Schmerzmedizin wurde durch die Ad-hoc-Kommission „Studienordnungen“ der Deutschen Gesellschaft zum Studium des Schmerzes e. V. bereits im Frühjahr 2008 ein erstes interdisziplinär erarbeitetes Kerncurriculum für die studentische Lehre im Rahmen der neuen Ärztlichen Approbationsordnung veröffentlicht [16].

Mit der Änderung der Ärztlichen Approbationsordnung im Sommer 2012 wurde ein neuer QSB 14 „Schmerzmedizin“ als Teil der curricularen Lehre in das Medizinstudium eingeführt. Durch diese Maßnahme sollte sichergestellt werden, dass zukünftig alle Medizinstudierenden zumindest eine theoretische und praktische Grundausbildung im Bereich der Schmerzmedizin erhalten. Die Medizinischen Fakultäten waren daher verpflichtet, dieses neue Fach zeitnah zu etablieren, um allen Studierenden die Möglichkeit zu geben, den entsprechenden Leistungsnachweis zu erlangen. Dieser war erstmalig für die Anmeldung zum 2. Abschnitt der Ärztlichen Prüfung im Herbst 2016 erforderlich.

Unklar blieb anfangs jedoch, wie und in welchem Umfang die Etablierung des neuen QSB vonstattengehen sollte, sodass es bei der Implementierung zu großen Unterschieden zwischen den verschiedenen medizinischen Fakultäten in Deutschland kam.

Die Hypothese der vorliegenden Untersuchung war, dass die Etablierung des QSB 14 „Schmerzmedizin“ an der Medizinischen Fakultät der Universität Leipzig in den folgenden Jahren zu einer Kompetenzsteigerung in der Selbsteinschätzung der Studierenden beitragen konnte.

## Studiendesign

### Das Leipziger Curriculum „Schmerzmedizin“

Die Etablierung des neuen QSB 14 wurde entsprechend den Empfehlungen zur schrittweisen Entwicklung eines Curriculums für medizinische Ausbildung nach Kern et al. realisiert [8]. Hierbei erfolgte zu Beginn eine Bedarfsanalyse der Zielgruppe der Studierenden mithilfe einer papierbasierten Umfrage. Die Befragung richtete sich an Studierende, die zu diesem Zeitpunkt noch keinen Kontakt zu Lehrinhalten der Schmerzmedizin hatten, und orientierte sich dabei an ähnlichen Befragungen anderer medizinischer Fakultäten [13, 22]. Ein vergleichbares Verfahren der Curriculumsentwicklung mit begleitender Evaluation wurde auch an der Universitätsmedizin Mainz beschrieben [17]. Nach Abschluss eines parallel stattfindenden curricularen Mappings mit der Suche nach Lehrveranstaltungen, die bereits schmerzmedizinische Inhalte vermitteln, wurde eine Arbeitsgruppe gegründet, die im Auftrag der Studienkommission die Implementierung des QSB begleiten sollte. Ausgehend von den Ergebnissen der Bedarfsanalyse und den bereits bestehenden Lehrveranstaltungen mit schmerzmedizinischem Fokus, priorisierte die Arbeitsgruppe die Lerninhalte und glich diese regelmäßig mit den deutschlandweit empfohlenen Inhalten ab [16]. Nach entsprechender Vorbereitung konnte das Curriculum beginnend mit dem Wintersemester 2014/2015 etabliert werden.

**Figure 1:**
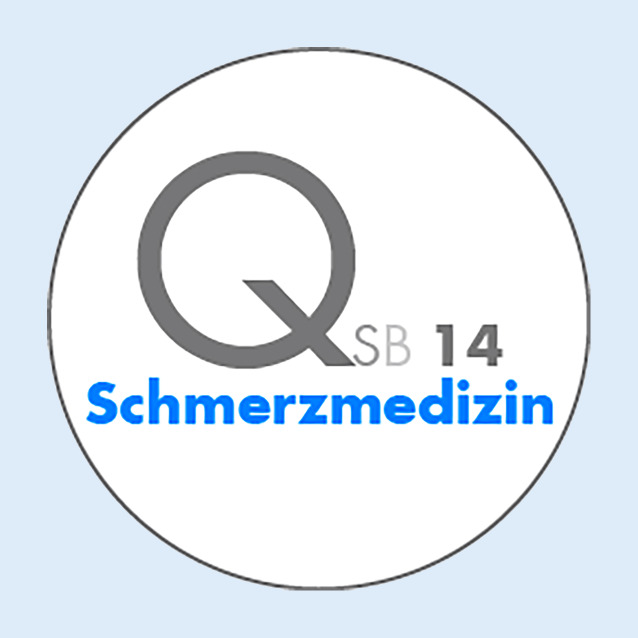

Das Curriculum erstreckt sich longitudinal über zwei Studienjahre vom 7. bis zum 10. Fachsemester und wurde zur Steigerung der Sichtbarkeit mit einem spezifischen Logo versehen (Abb. 1).

Im Curriculum sind insgesamt vier praktische Kurse am Krankenbett von jeweils 90 min Dauer vorgesehen. Die Kurse in der Anästhesiologie und in der Pädiatrie sind Teil des 4. Studienjahrs. Ergänzt werden diese Lehrveranstaltungen durch zwei weitere Praktika im 5. Studienjahr im Bereich der Neurologie und der Psychosomatik. Eine Übersicht über die Inhalte der Praktika gibt Tab. 1.

**Table 1:** 

Praktikum Anästhesiologie	Grundlegende Aspekte einer gezielten Schmerzanamnese
	Grundlegende Konzepte der medikamentösen Schmerztherapie anhand des WHO-Stufenschemas
	Grundlagen der multimodalen Schmerztherapie
	Begleitung des Akutschmerzdiensts bei Visite auf den operativen Stationen
	Demonstration von PCA- und PCEA-Systemen
Praktikum Pädiatrie	Besonderheiten der Schmerzanamnese und Untersuchung bei Kindern und Jugendlichen
	Diskussion verschiedener Szenarien anhand von Fallbeispielen oder Patienten (nach Verfügbarkeit)
Praktikum Neurologie	Gezielte Anamnese zu Kopfschmerzen und neuropathischen Schmerzen am Patienten
	Spezielle und gezielte Untersuchungstechniken
	Festlegung einer zielgerichteten Therapie
	Ggf. Vorstellung weiterer schmerzmedizinisch relevanter Krankheitsbilder je nach Verfügbarkeit
Praktikum Psychosomatik	Gezielte Anamnese eines chronischen Schmerzpatienten unter psychosomatischen Gesichtspunkten
	Nachbesprechung und Diskussion einer möglichen bedarfsgerechten Therapie bei psychosomatisch erkrankten Patienten mit chronischen Schmerzen

*PCA* „patient-controlled analgesia“, *PCEA* „patient-controlled epidural analgesia“

Für Studierende im zehnten Fachsemester findet eine interdisziplinäre Vorlesungsreihe mit insgesamt acht Vorlesungen á 45 min statt. Hierbei werden physiologische und pharmakologische Grundlagen der Schmerzmedizin wiederholt als auch die Themen der Praktika nochmals aufbereitet und um weitere Aspekte wie beispielsweise schmerztherapeutische Verfahren in der Neurochirurgie ergänzt. Die Inhalte der Vorlesung wurden den Studierenden ergänzend auch in Form digitaler Handreichungen sowie als digitale Aufzeichnungen (Video-Podcasts) zugangsgeschützt zur Verfügung gestellt. Die Vorlesungsreihe schließt mit einer Multiple-Choice-Klausur ab, deren Bestehen neben der erfolgreichen Teilnahme an den Praktika Grundvoraussetzung für den Erwerb des benoteten Leistungsnachweises ist. Einzelheiten zu den Inhalten der Vorlesungsreihe zeigt Tab. 2.

**Table 2:** 

	Thema und Vorlesungsinhalte	Zuständigkeit
1	*Grundlagen der Schmerztherapie*	Anästhesiologie
	– Definition von Schmerzen sowie ethische und rechtliche Grundlagen der Schmerzmedizin („Das Recht auf Schmerzbehandlung“)	
	– Klassifikation von Schmerzen (akut vs. chronisch; nozizeptiv vs. neuropathisch)	
	– Typische Komorbiditäten von Schmerzen	
	– Allgemeine Schmerzanamnese und Messinstrumente für Schmerzen	
	– Grundprinzipien der Schmerzbehandlung (kausal vs. symptomatisch und pharmakologisch vs. nichtpharmakologisch)	
2	*(Patho‑)Physiologie und Pharmakotherapie in der Schmerzmedizin*	Pharmakologie
	– Physiologie von Schmerzen (Entstehung, Weiterleitung und Verarbeitung von Schmerzsignalen)	
	– Pharmakologische Grundlagen von Opioiden und Nichtopioidanalgetika	
	– Pharmakologische Grundlagen zu typischen Koanalgetika	
3	*Akutschmerztherapie und Tumorschmerztherapie*	Anästhesiologie
	– Postoperativer und posttraumatischer Schmerz	
	– Praktische Beispiele und Behandlungsschemata für operative Eingriffe mit geringen, mittelstarken und starken postoperativen Schmerzen	
	– Praktische Anwendung des WHO-Stufenschemas im Rahmen der Tumorschmerztherapie	
	– Grundlagen zur Rezeptierung von Opioiden nach der BtMVV	
4	*Schmerzchronifizierung, neuropathische Schmerzen und multimodale Schmerztherapie*	Anästhesiologie
	– Therapie chronischer Schmerzen am Beispiel des chronischen nichtspezifischen Rückenschmerzes	
	– Möglichkeiten der invasiven und der nichtpharmakologischen Schmerztherapie (Trainingstherapie, Thermo‑/Elektrotherapie, Akupunktur, …)	
	– Grundlagen der Diagnostik und Therapie neuropathischer Schmerzen am Beispiel der Polyneuropathie und der Zosterneuralgie	
	– Wichtige Aspekte der multimodalen Schmerztherapie	
5	*Schmerztherapie in der Pädiatrie*	Pädiatrie
	– Besonderheiten der Schmerzanamnese im Kindes- und Jugendalter	
	– Vorstellung typischer Analgetika in der Pädiatrie anhand beispielhafter Behandlungsschemata	
	– Besonderheiten der Pharmakotherapie in der Pädiatrie (Dosierung nach Körpergewicht, „off label use“ der Medikamente, Indikation für unterschiedliche Applikationswege, Tageshöchstdosen)	
6	*Kopfschmerz*	Neurologie
	– Diagnostik und Behandlung von Migräne‑/Clusterkopfschmerzen sowie Kopfschmerz bei Medikamentenabusus	
7	*Psychosomatische Schmerzkonzepte*	Psychosomatik
	– Psychosoziale Zusammenhänge der Schmerzentstehung und der Schmerzunterhaltung (affektive Faktoren, kognitive und Verhaltensfaktoren)	
	– Psychiatrische und psychosomatische Komorbidität, soziale Einflüsse	
8	*Schmerztherapeutische Verfahren in der Neurochirurgie*	Neurochirurgie
	– Vorstellung neurochirurgischer Therapiemöglichkeiten bei chronischen Schmerzen	
	– Fallbeispiele (u. a. Spinal-cord-Stimulation)	

*BtMVV* Betäubungsmittel-Verschreibungsverordnung

### Befragungen und Datenauswertung

Die Befragungen im Rahmen der Untersuchung erfolgten zu drei Zeitpunkten. Eine erste Befragung der Studierenden wurde zum Ende des Sommersemesters 2014 vor Eintritt in das Praktische Jahr durchgeführt zur Erfassung des Ausgangszustands. In einem zweiten Schritt wurden zum Ende des Sommersemesters 2016 die Studierenden befragt, die das neu etablierte Curriculum erstmalig vollständig durchlaufen hatten. Abschließend erfolgte nach kontinuierlichen Evaluationen und damit verbundenen Anpassungen eine weitere Befragung fünf Jahre nach Start des QSB am Ende des Sommersemesters 2019.

Die Fragebögen waren jeweils identisch und bestanden aus drei Teilen, wobei sie sich inhaltlich an zwei zu diesem Zeitpunkt bereits publizierten Befragungen orientierten [13, 22]. Neben einem allgemeinen Teil zur Bewertung des Lehrangebots gab es einen zweiten Teil, der den subjektiven Wissensstand der Studierenden abfragte. In einem dritten Teil wurden die Studierenden nach ihrer Einschätzung zur klinischen Relevanz bestimmter schmerzmedizinischer Lehrinhalte befragt. Die Erfassung erfolgte dabei jeweils mithilfe 5‑stufiger Likert-Skalen. Der vollständige Fragebogen ist im Online-Zusatzmaterial enthalten, wobei die enthaltenen Fragen auch in den Ergebnissen einsehbar sind.

Die Abfragen wurden mithilfe der Software EvaSys (evasys GmbH, Lüneburg, Deutschland) durchgeführt. Die Befragung im Sommersemester 2014 erfolgte online – die anderen beiden Befragungen papierbasiert. Die papierbasierten Fragebögen wurden digital eingelesen und manuell verifiziert. Die statistische Auswertung und die Erstellung der Grafiken erfolgten mithilfe der Software GraphPad Prism (GraphPad Software, LLC; San Diego, CA, USA). Die Testung auf Signifikanzunterschiede zwischen den Befragungszeitpunkten erfolgte mithilfe des Kruskal-Wallis-Tests. Das Signifikanzniveau wurde auf *p* < 0,05 festgelegt.

## Ergebnisse

An der Onlinebefragung im Sommersemester 2014 haben 32 Studierende teilgenommen (Rücklaufquote 10 %). An den papierbasierten Befragungen im Sommersemester 2016 haben 227 Studierende (Rücklaufquote 71 %) und im Sommersemester 2019 haben 60 Studierende (Rücklaufquote 19 %) teilgenommen.

**Figure 2:**
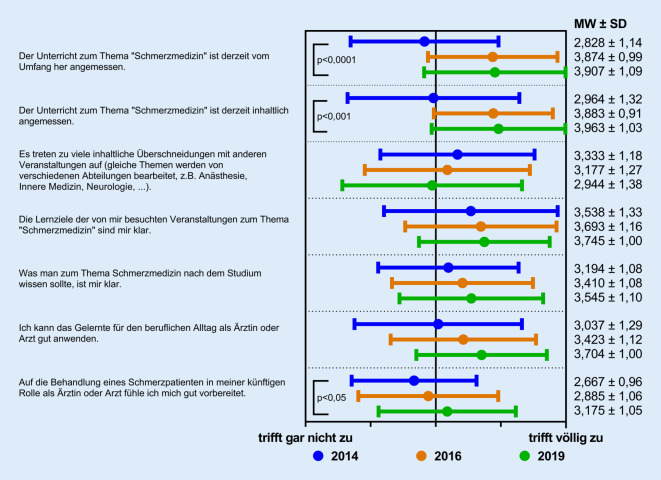

Die Befragungsergebnisse für den allgemeinen Teil, in dem es um eine übergreifende Bewertung des QSB und der Bedeutung für den klinischen Alltag geht, sind in Abb. 2 dargestellt.

Signifikante Verbesserungen nach Einführung des QSB gab es bei der Bewertung auf der 5‑stufigen Likert-Skala („trifft gar nicht zu“ [Wert ≙ 1] bis „trifft völlig zu“ [Wert ≙ 5]) bezüglich des Umfangs des Unterrichts zum Thema Schmerzmedizin (von 2,828 ± 1,14 [MW ± SD] auf 3,907 ± 1,09 [*p* < 0,0001]) sowie der Angemessenheit der vermittelten Inhalte (von 2,964 ± 1,32 auf 3,963 ± 1,03 [*p* < 0,001]). Ebenfalls eine signifikante Verbesserung gab es bei der Frage, inwieweit sich die Studierenden auf die künftige Behandlung eines Schmerzpatienten vorbereitet fühlen (2,667 ± 0,96 auf 3,175 ± 1,05 [*p* < 0,05]).

Die Befragungen ergaben eine hohe Vigilanz der Studierenden für die Relevanz schmerzmedizinischer Inhalte im Studiengang. Fast alle abgefragten Items wurden mit mindestens „wichtig“ bewertet – dies galt auch bereits vor Etablierung des QSB. Die durchschnittlich höchsten Bewertungen bekamen hierbei die Items „Erhebung einer Schmerzanamnese“, „Durchführung einer Schmerz-Symptom-orientierten körperlichen Untersuchung“ sowie „Diagnose und Therapie von akuten Schmerzen“. Als am wenigsten wichtig – aber immer noch mit einer Bewertung zwischen „neutral“ und „wichtig“ – wurde der Themenbereich „Komplementärmedizinische Therapieansätze für Schmerzpatienten“ bewertet.

**Figure 3:**
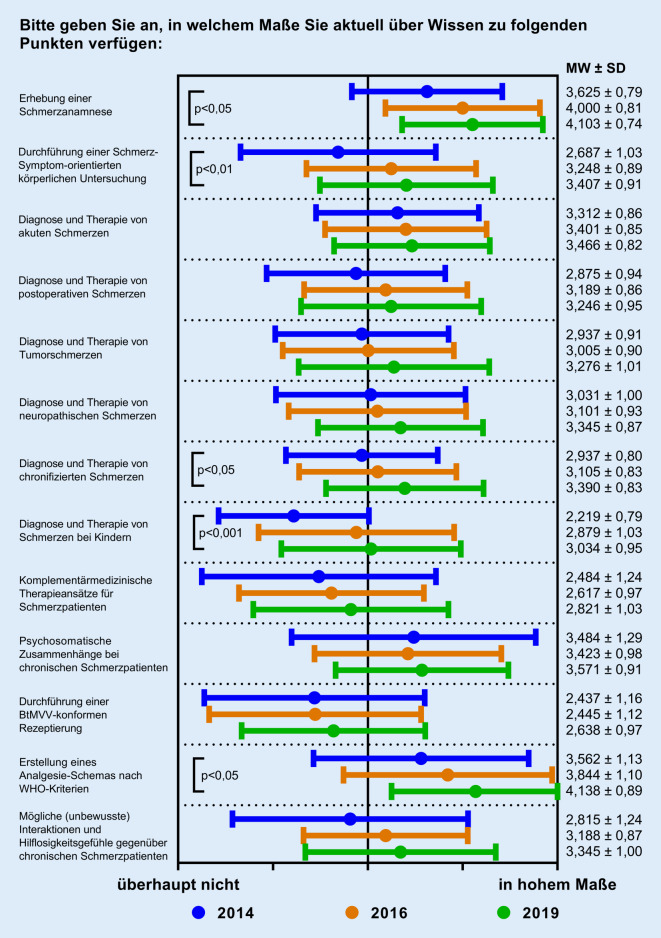

Eine Selbsteinschätzung der Studierenden bezüglich ihres aktuellen Wissensstands hinsichtlich verschiedener Kompetenzen auf einer 5‑stufigen Likert-Skala („überhaupt nicht“ [Wert ≙ 1] bis „in hohem Maße“ [Wert ≙ 5]) zu den verschiedenen Befragungszeitpunkten zeigt Abb. 3. Die Etablierung des QSB führte bei nahezu allen Punkten zu einer stetigen Verbesserung, die bei fünf der abgefragten Aspekte auch signifikant war. Hierzu zählten die „Erhebung einer Schmerzanamnese“ (von 3,625 ± 0,79 auf 4,103 ± 0,74 [*p* < 0,05]), die „Durchführung einer Schmerz-Symptom-orientierten körperlichen Untersuchung“ (von 2,687 ± 1,03 auf 3,407 ± 0,91 [*p* < 0,01]), die „Diagnose und Therapie von chronischen Schmerzen“ (von 2,937 ± 0,80 auf 3,390 ± 0,83 [*p* < 0,05]), die „Diagnose und Therapie von Schmerzen bei Kindern“ (von 2,219 ± 0,79 auf 3,034 ± 0,95 [*p* < 0,001]) und die „Erstellung eines Analgesie-Schemas nach WHO-Kriterien“ (von 3,562 ± 1,13 auf 4,138 ± 0,89 [*p* < 0,05]).

## Diskussion

Die Ergebnisse dieser Erhebung zeigen, dass die Studierenden vor Einführung eines QSB für Schmerzmedizin ein relevantes Defizit im Bereich der schmerzmedizinischen Lehre wahrgenommen haben. Nach Einführung des neuen QSB kam es hier zu einer signifikanten Verbesserung, wobei sowohl der Umfang als auch die vermittelten Inhalte mehrheitlich als angemessen wahrgenommen werden.

Infolge der Evaluation konnten in den ersten Semestern vor allem inhaltliche Überschneidungen in den Vorlesungen abgebaut werden, wodurch es gelang, die begrenzte Stundenanzahl möglichst effektiv zu nutzen. Neben der regelmäßigen Evaluation ist die Verbesserung mutmaßlich auch auf die zunehmend konkreteren Vorgaben von Lernzielen und den Abgleich mit dem Prüfungswissen im Sinne eines Blueprints zurückzuführen [28]. Die Anpassung der Vorgaben erfolgte dabei in regelmäßigen Lehrkonferenzen der beteiligten Fächer zu Beginn eines Semesters. Dies hatte auch Auswirkungen auf die Praktika, die in der Folge – unabhängig von den jeweils Lehrenden – immer standardisierter wurden, um einerseits alle geforderten Inhalte abzudecken und gleichzeitig auch für eine konstant hohe Lehrqualität zu sorgen. Bei aller Motivation, die zur Verfügung stehende Zeit möglichst maximal mit neuen Informationen zu füllen, sollte man jedoch berücksichtigen, dass eine Wiederholung wichtiger Inhalte aus lerntheoretischer Sicht sinnvoll ist [12].

Auch wenn es bei der Frage, ob man sich auf die Behandlung von Schmerzpatienten gut vorbereitet fühlt, zu einer signifikanten Verbesserung gekommen ist, ist das Ergebnis insgesamt eher ernüchternd. Die Frage war hier bewusst allgemein gehalten. Eventuell sind stärkere Änderungen in der Selbsteinschätzung zu erwarten, wäre eine Unterscheidung nach Akutschmerztherapie, chronischer Schmerztherapie, Schmerztherapie im Kindesalter usw. erfolgt. Eine Verzerrung der Ergebnisse durch besonders engagierte oder frustrierte Studierende zu den Umfragezeitpunkten 2014 und 2019 kann nicht ausgeschlossen werden.

Auch weltweit besteht der Anhalt, dass schmerzmedizinische Inhalte in den Curricula im Vergleich zur Versorgungsrealität unterrepräsentiert sind [6, 25]. Unsere Ergebnisse zeigen, dass den Studierenden die Bedeutung schmerzmedizinischer Lehrinhalte dabei sehr wohl bewusst ist und es von ihnen als wichtig erachtet wird, am Ende des Studiums über entsprechendes Wissen zu verfügen.

Eine Limitation der Befragung ist die zu den verschiedenen Befragungszeitpunkten unterschiedliche und zu zwei Zeitpunkten geringe Rücklaufquote, die eine statistische Analyse und die Aussagekraft der Ergebnisse einschränkt. Organisatorisch konnte die Befragung jedoch nur zu einem Zeitpunkt (2016) als papierbasierte Befragung mit der Klausur gekoppelt werden, sodass hier mit 71 % die höchste Rücklaufquote erreicht wurde. Die Befragung mit der geringsten Rücklaufquote erfolgte 2014 online. Eine papierbasierte Befragung war zu diesem Zeitpunkt kurz vor Beginn des Praktischen Jahrs nicht möglich, da viele Studierende bereits nicht mehr am Studienort verfügbar waren. Die papierbasierte Folgebefragung 2016 führte zu deutlich höheren Rücklaufquoten, insbesondere da die Fragebögen hier parallel mit der Abschlussklausur des QSB verteilt werden konnten. 2019 war diese Kopplung leider nicht möglich, da die Abschlussklausur zu einem früheren Zeitpunkt im Semester angesetzt wurde und somit ein relevanter Teil der Studierenden noch nicht alle praktischen Kurse absolviert hatte. Die Fragebögen wurden daher am Semesterende verteilt, wobei die Rücklaufquote leider wieder deutlich niedriger ausfiel. Zur Erlangung einer hohen Rücklaufquote sollten die Befragungen künftig also weiterhin papierbasiert und möglichst zusammen mit der dazugehörigen Abschlussprüfung oder letzten Lehrveranstaltung des Fachgebiets erfolgen.

Auf der Basis der Ergebnisse lässt sich die Frage formulieren, wie man die Kompetenzen der Studierenden künftig noch weiter verbessern kann. Eine Möglichkeit wäre die nochmalige bzw. weitere Anpassung des Curriculums. Hier sollte es ein Ziel sein, einen noch stärkeren Fokus auf die Erlangung von Kompetenzen zu legen und dabei noch differenzierter zwischen den verschiedenen zu erreichenden Kompetenzebenen zu unterscheiden. So kann bei einigen Lernzielen am Ende des Studiums das reine „Wissen“ ausreichend sein, während bei anderen Aspekten eine Handlungskompetenz der Studierenden anzustreben ist. Eine Orientierung kann hierbei die Neuauflage des Nationalen Kompetenzbasierten Lernzielkatalogs (NKLM) sein, die vor wenigen Monaten veröffentlicht wurde und für die aktuell zudem Fächerempfehlungen für die Vermittlung einzelner Lernziele ergänzt wurden [20]. Hierbei gilt es, Schmerz auch als eine potenziell unabhängige Erkrankung und nicht mehr nur als reines Symptom zu betrachten. Dabei sollten neben der Kompetenz in der körperlichen Untersuchung, der Verschreibung von Medikamenten usw. auch Empathie, Kommunikation und soziale/psychologische Komponenten in Diagnostik und Therapie in einem multiprofessionellen Ansatz verstärkt vermittelt werden [5, 7, 19]. Ziel muss es sein, dass die Studierenden hinsichtlich der zu erwartenden Versorgungsrealität effektiv vorbereitet werden. Dies bedeutet auch, das Bewusstsein der Studierenden für das Risiko und die Möglichkeiten zur Vermeidung von Schmerzchronifizierung zu steigern [1, 23, 29].

Eine weitere Möglichkeit zur Optimierung des QSB wäre eine Anpassung in der Art der Wissensvermittlung. Neben der reinen Vermittlung von Faktenwissen durch Vorlesungen bzw. Anwendungs- und Begründungswissen in Seminaren, könnte hier vor allem der praktische Unterricht am Krankenbett gestärkt werden. Die Ergebnisse der vorliegenden Befragung zeigen, dass die Lernziele, die den größten und signifikanten Zuwachs hatten, alle im Rahmen des praktischen Unterrichts am Krankenbett vermittelt wurden.

Ein Punkt, der im Aufbau des aktuellen Curriculums kritisch beleuchtet werden sollte, ist die Vermittlung der kognitiven Lernziele im Rahmen der Vorlesungsreihe im 10. Fachsemester und somit nach bzw. parallel zu den Praktika im 4. und 5. Studienjahr. Prinzipiell erscheint es sinnvoll, die Praktika und damit die Vermittlung der Handlungskompetenz erst nach der Vorlesungsreihe anzusetzen [11]. Dies ist jedoch im aktuellen Gesamtkonzept des hiesigen Medizinstudiums nicht umsetzbar, da die einzelnen Praktika zur Schmerzmedizin mit den sonstigen Praktika der Fächer (Anästhesiologie, Pädiatrie usw.) verknüpft sind und es für die Vorlesungsreihe in den anderen Semestern während der Etablierung keinen anderen verfügbaren Zeitpunkt gab. Dieser späte Zeitpunkt bietet jedoch den Vorteil, dass auf die Diagnostik und Therapie schmerzrelevanter Krankheitsbilder Bezug genommen werden kann, die in den bereits abgeschlossenen Vorlesungsreihen der Neurologie, Orthopädie usw. besprochen wurden. So kann dem interdisziplinären Charakter der Schmerzmedizin noch einmal gesondert Ausdruck verliehen werden.

Im Rahmen der praktischen Ausbildung könnte der Einsatz von Simulationspersonen zur weiteren Kompetenzsteigerung der Studierenden beitragen [18, 27], vor allem im Bereich der Anamneseerhebung, der körperlichen Untersuchung und im Rahmen von Beratungsgesprächen. Hierfür sind jedoch fortlaufende Kosten zur Bereitstellung von Simulationspersonen zu berücksichtigen.

Digitale Lehreinheiten könnten zudem ressourcenschonend wiederholt eingesetzt werden und Studierenden darüber hinaus die Möglichkeit zum selbstgesteuerten Lernen bieten. Nicht zuletzt bedingt durch die SARS-CoV-2-Pandemie wurden in diesem Bereich große Fortschritte erzielt, wobei es nun gilt, die erlangte Expertise zu verstetigen [15, 21]. Zukünftig wird es daher das Ziel sein, neben der dringend notwendigen Präsenzlehre auch weiterhin ergänzende und vertiefende Onlineangebote im Selbststudium bereitzustellen. So ist für unseren Standort beispielsweise die Erstellung einer Onlinelehreinheit zum Thema „Durchführung einer BtMVV-konformen Rezeptierung“ geplant, um die in den Ergebnissen der hier vorliegenden Evaluation ersichtlichen Kompetenzdefizite zeitnah adressieren zu können.

Ein weiterer Aspekt, der gerade auch im Hinblick auf die multimodale Schmerztherapie Relevanz hat, ist die Stärkung der interprofessionellen Ausbildung [2, 3, 10]. Neben gemeinsamen Lehreinheiten mit Pflegekräften sind hier auch Verknüpfungen mit Vertreterinnen und Vertretern der Psychologie, Physiotherapie, Sozialarbeit, Pharmazie und weiterer Disziplinen denkbar, wobei die Bereitstellung der dafür notwendigen personellen und finanziellen Ressourcen eine Herausforderung bleibt.

Einen weiteren wichtigen Aspekt für den Wissenserwerb stellen Prüfungen dar. Ganz im Sinne von „assessment drives learning“ wird vor allem das gelernt, was geprüft wird. Die Selbsteinschätzung bzw. -reflexion des Wissensstands der Studierenden kann hier ein erster Hinweis sein – letztlich sind aber valide, standardisierte Prüfungen notwendig [4, 24]. Wichtig ist es, die Prüfungsformate so anzupassen, dass auch Kompetenzen geprüft werden können [26, 28]. Hierfür ist die Integration schmerzmedizinischer Lehrinhalte in OSCE-Prüfungen (Objective Structured Clinical Examination), z. B. unter Zuhilfenahme von Simulationspersonen, oder die Entwicklung arbeitsplatzbasierter Prüfungen denkbar. Die Erstellung einer OSCE-Prüfung rein für den QSB erscheint in unseren Augen nicht sinnvoll. Vielmehr sollte die Integration schmerzmedizinischer Inhalte in eine zu entwickelnde interdisziplinäre OSCE vor Beginn des Praktischen Jahrs angestrebt werden, wie sie entsprechend dem Entwurf der neuen Ärztlichen Approbationsordnung von den medizinischen Fakultäten künftig gefordert wird. Auf diese Weise kann man den Aufwand für die einzelnen Fachgebiete begrenzen und vor allem auch der hohen Interdisziplinarität des Fachgebiets Rechnung tragen. Die aktuell weiter stattfindende Überarbeitung der Ärztlichen Approbationsordnung wird zeigen, inwieweit dies künftig auch in den Staatsexamensprüfungen selbst Berücksichtigung findet [9, 14].

Für die Zukunft werden wir uns in den kommenden Jahren an der weiteren Entwicklung des NKLM und der Ärztlichen Approbationsordnung orientieren. Neben einem reinen Mapping der Lernziele des NKLM und einem Abgleich mit den Fächerempfehlungen steht hier vor allem der Umbau des Leipziger Studiengangs hin von einem Regelstudium zu einem integrativen Z‑Curriculum im Fokus. Hierbei gilt es, den Fachbereich der Schmerzmedizin auch weiterhin longitudinal abzubilden. Neben der dann longitudinal durchgeführten wiederholten Überprüfung von Lerninhalten sollen hier auch die Integration neuer Lehrformate und die Integration der Expertise zusätzlicher Fachgebiete zu einer weiteren Kompetenzsteigerung beitragen.

## Fazit für die Praxis

- Die Einführung des QSB „Schmerzmedizin“ hat an der Universität Leipzig zu einer Verbesserung in der Selbsteinschätzung der Studierenden geführt.
- Ziel muss es sein, das Curriculum noch stärker auf die Vermittlung von Kompetenzen zu fokussieren. Dabei sind sowohl die künftige Versorgungsrealität der Absolvierenden als auch die Vorgaben des neuen NKLM zu berücksichtigen. Hierbei sollte bei der Konzeption und Überarbeitung der Lehrveranstaltungen soweit möglich ein multiprofessioneller Ansatz berücksichtigt werden.
- Zukünftig sollen darüber hinaus digitale Lehreinheiten und Simulationen für die standardisierte Vermittlung praktischer Kompetenzen verstärkt integriert werden.
- Das Mapping gegen den NKLM 2.0 und die darin enthaltenen Fächerempfehlungen werden zeigen, ob ggf. gänzlich neue Inhalte künftig mit in den QSB integriert werden sollten.
- Insbesondere für praktische Kompetenzen sollten auch praktische Prüfungsformate wie arbeitsplatzbasierte Prüfungen und OSCE verwendet werden.

## Supplementary Information




