# Supplementary material for: The cross-sectional field “pain medicine” in medical studies at the University of Leipzig—What has been achieved? An analysis of self-estimation of students before, during and 5 years after establishment of the cross-sectional field
Source: Schmerz. 2022 Aug 26;36(6):389–97. [Article in German] doi: 10.1007/s00482-022-00665-7 (PMC9415254; doi:10.1007/s00482-022-00665-7)
Supplement: Supplementary file 1 [file 482_2022_665_MOESM1_ESM.pdf]

Online-Zusatzmaterial

Der QSB 14 „Schmerzmedizin“ an der Universität Leipzig – was wurde erreicht?

Eine Analyse der Selbsteinschätzung der Studierenden vor, während und fünf Jahre nach Etablierung des Querschnittsbereiches

Gunther Hempel[1]; Andreas Weissenbacher[1]; Diana Becker-Rux[1]; Swantje Mescha[1,2]; Sebastian N. Stehr[1], Robert Werdehausen[1]

[1] Klinik und Poliklinik für Anästhesiologie und Intensivtherapie; Universitätsklinikum Leipzig AöR; Leipzig, Deutschland

[2] Klinik für Anästhesiologie und Intensivmedizin; Universitätsklinikum Jena; Jena, Deutschland

Allgemeiner Teil

	trifft gar nicht zu				trifft völlig zu	weiß nicht
Der Unterricht zum Thema "Schmerzmedizin" ist derzeit vom Umfang her angemessen.	☐	☐	☐	☐	☐	☐
Der Unterricht zum Thema "Schmerzmedizin" ist derzeit inhaltlich angemessen.	☐	☐	☐	☐	☐	☐
Es treten zu viele inhaltliche Überschneidungen mit anderen Veranstaltungen auf (gleiche Themen werden von verschiedenen Abteilungen bearbeitet, z. B. Anästhesie, Innere Medizin, Neurologie, ...).	☐	☐	☐	☐	☐	☐
Die Lernziele der von mir besuchten Veranstaltungen zu Thema "Schmerzmedizin" sind mir klar.	☐	☐	☐	☐	☐	☐
Was man zum Thema Schmerzmedizin nach dem Studium wissen sollte, ist mir klar.	☐	☐	☐	☐	☐	☐
Ich kann das Gelernte für den beruflichen Alltag als Ärztin oder Arzt gut anwenden.	☐	☐	☐	☐	☐	☐
Auf die Behandlung eines Schmerzpatienten in meiner künftigen Rolle als Ärztin oder Arzt fühle ich mich gut vorbereitet.	☐	☐	☐	☐	☐	☐

Spezieller Teil

Bitte beachten Sie, dass sich die Beschriftung der Skalen im folgenden speziellen Teil vom allgemeinen Teil unterscheidet ("in hohem Maße" links und "überhaupt nicht" rechts).

Bitte geben Sie an, in welchem Maße Sie *aktuell über Wissen* zu folgenden Punkten verfügen.

	in hohem Maße				überhaupt nicht	weiß nicht
Erhebung einer Schmerzanamnese	☐	☐	☐	☐	☐	☐
Durchführung einer Schmerz-Symptom-orientierten körperlichen Untersuchung	☐	☐	☐	☐	☐	☐
Diagnose und Therapie von akuten Schmerzen	☐	☐	☐	☐	☐	☐
Diagnose und Therapie von postoperativen Schmerzen	☐	☐	☐	☐	☐	☐
Diagnose und Therapie von Tumorschmerzen	☐	☐	☐	☐	☐	☐
Diagnose und Therapie von neuropathischen Schmerzen	☐	☐	☐	☐	☐	☐
Diagnose und Therapie von chronifizierten Schmerzen	☐	☐	☐	☐	☐	☐
Diagnose und Therapie von Schmerzen bei Kindern	☐	☐	☐	☐	☐	☐
Komplementärmedizinische Therapieansätze für Schmerzpatienten	☐	☐	☐	☐	☐	☐
Psychosomatische Zusammenhänge bei chronischen Schmerzpatienten	☐	☐	☐	☐	☐	☐
Durchführung einer BtMVV-konformen Rezeptierung	☐	☐	☐	☐	☐	☐
Erstellung eines Analgesie-Schemas nach WHO-Kriterien	☐	☐	☐	☐	☐	☐
Mögliche (unbewusste) Interaktionen und Hilflosigkeitsgefühle gegenüber chronischen Schmerzpatienten	☐	☐	☐	☐	☐	☐

Abbildung 1: Übersicht des Fragebogens zur Datenerfassung - Seite 1

Spezieller Teil [Fortsetzung]

<u>Für wie wichtig halten Sie es, *am Ende des Studiums* Wissen über folgende Punkte zu haben.</u>

	**sehr wichtig**				**gar nicht wichtig**	**weiß nicht**
Erhebung einer Schmerzanamnese	☐	☐	☐	☐	☐	☐
Durchführung einer Schmerz-Symptom-orientierten körperlichen Untersuchung	☐	☐	☐	☐	☐	☐
Diagnose und Therapie von akuten Schmerzen	☐	☐	☐	☐	☐	☐
Diagnose und Therapie von postoperativen Schmerzen	☐	☐	☐	☐	☐	☐
Diagnose und Therapie von Tumorschmerzen	☐	☐	☐	☐	☐	☐
Diagnose und Therapie von neuropathischen Schmerzen	☐	☐	☐	☐	☐	☐
Diagnose und Therapie von chronifizierten Schmerzen	☐	☐	☐	☐	☐	☐
Diagnose und Therapie von Schmerzen bei Kindern	☐	☐	☐	☐	☐	☐
Komplementärmedizinische Therapieansätze für Schmerzpatienten	☐	☐	☐	☐	☐	☐
Psychosomatische Zusammenhänge bei chronischen Schmerzpatienten	☐	☐	☐	☐	☐	☐
Durchführung einer BtMVV-konformen Rezeptierung	☐	☐	☐	☐	☐	☐
Erstellung eines Analgesie-Schemas nach WHO-Kriterien	☐	☐	☐	☐	☐	☐
Mögliche (unbewusste) Interaktionen und Hilflosigkeitsgefühle gegenüber chronischen Schmerzpatienten	☐	☐	☐	☐	☐	☐

Persönliche Anmerkungen

Welche Anregungen, Verbesserungsvorschläge und/oder Kritik möchten Sie uns mitteilen?

Abbildung 2: Übersicht des Fragebogens zur Datenerfassung - Seite 2
